# Percutaneous coronary intervention for chronic total occlusion in octogenarians: a propensity score study

**DOI:** 10.1038/s41598-022-06994-y

**Published:** 2022-02-23

**Authors:** Recha R. L. Blessing, Majid Ahoopai, Martin Geyer, Moritz Brandt, Andreas M. Zeiher, Mariuca Vasa-Nicotera, Thomas Münzel, Philip Wenzel, Tommaso Gori, Zisis Dimitriadis

**Affiliations:** 1 Department of Cardiology, University Medical Center Mainz - Center of Cardiology, Johannes Gutenberg University, Langenbeckstr.1, 55131 Mainz, Germany; 2 German Center for Cardiovascular Research (DZHK), Partner Site Rhine-Main, Mainz, Germany; 3 Department of Cardiology, Center of Internal Medicine, Goethe University Frankfurt, Theodor-Stern-Kai 7, 60590 Frankfurt, Germany; 4 Center for Thrombosis and Hemostasis (CTH), Johannes Gutenberg University, Mainz, Germany

**Keywords:** Interventional cardiology, Ischaemia

## Abstract

Feasibility and efficacy of complex percutaneous coronary intervention (PCI) in the elderly, a more frail population due to more comorbidities is incompletely understood. We therefore set out to compare success and complication rate of PCI for chronic total occlusion (CTO) in octogenarians, in comparison to non-octogenarians. Data from 267 patients (58 patients over 80 years of age and 209 under 80 years of age) who had undergone CTO PCI were analyzed. To compare the results we calculated the propensity score and used inverse probability of treatment weighting. We evaluated demographic, clinical, angiographic, and periprocedural information. The median age of the total collective was 68 (31–90) years (octogenarian collective 82 (80–90) years vs non-octogenarians 65 (31–79) years). We observed a high success rate in both collectives (82.8% vs 90.4%, *p* = 0.10) and no difference in periprocedural complications or complications in the follow-up period. In our collective restenosis rate at follow-up was comparable to the propensity sore weighted population (11.3% vs 16.3%, *p* = 0.9). Our results show that CTO PCI in older patients is safe and feasible with comparable in-hospital and follow-up complication rates compared to a younger patient population.

## Introduction

Although there has been remarkable progress in the treatment of coronary artery disease (CAD), the disease continues to be the leading cause of morbidity and mortality worldwide. Elderly patients are predominantly affected by CAD. Among the classic cardiovascular risk factors like hypertension, diabetes mellitus, smoking or hypercholesterolemia, increasing age is the strongest and only one that obviously cannot be modulated by medical therapy. The demographic change is an increasing challenge of modern societies and especially treatment of CAD in elderly people is becoming more and more important^1^. Treatment of elderly patients with CAD is often challenging due to higher prevalence of comorbidities and complex coronary lesions as well as higher risk for procedural complications.

About 15–20% of all patients with CAD have a chronic total occlusion (CTO) of a coronary artery. Advances in catheter techniques, materials and treatment algorithms have increased the success rate of CTO PCI^2^.

Currently, clinical evidence proving advantages after successful CTO recanalization like a reduction in mortality, improvement of left ventricular function or quality of life are almost exclusively based on cohorts of younger patients with less comorbidities^2–4^, whereas data on possible benefits of the treatment in elderly patients are scarce. Thus, older patients with CTO are less likely to receive interventional treatment and are often treated with optimal drug therapy (OMT) alone^4–7^. Only few studies have investigated whether patients older than 75 years may benefit from interventional CTO recanalization^8–15^. To the best of our knowledge, the collective of patients 80 years of age or older has not been analyzed yet.

The aim of our study was to investigate the feasibility of CTO recanalization, occurrence of complications and restenosis in a patient collective of octogenarians compared to non-octogenarians.

## Materials and methods

### Study design and study population

The monocentric CTO PCI database of the University Medical Center in Mainz, Germany was retrospectively analyzed for patients that consecutively underwent recanalization of a CTO lesion and stratified for age (younger vs older than 80 years). The database included demographic, clinical, angiographic, and periprocedural information, along with in-hospital and in part long-term outcomes. In total, data from 267 patients who had undergone revascularization of a CTO from April 2016 to May 2021 were enrolled. Thirty-one (11.6%) patients had an acute coronary syndrome 236 (88.4%) chronic coronary syndrome. The patients presented with an acute coronary syndrome (STEMI-ACS or NSTEMI-ACS) underwent an initial coronary angiography and treatment of the culprit lesion. Diagnosed CTO lesions were treated in a staged procedure and after proof of myocardial vitality was generated by MRI in 16.8% of the patients, the other patients of our collective underwent stress-echocardiography.

Coronary angiography was performed by experienced operators. The CTO hybrid algorithm^16^ was used in all cases, starting with antegrade approaches and in case of failure escalation in retrograde approach. For bilateral injection we used two access sites, a combination of femoral and radial access were preferred over double femoral access whenever possible. Second generation drug eluting stents were implanted in all of the patients. Recommendations regarding antiplatelet regime after intervention were carried out in adherence to the guidelines^17,18^. Surveillance angiography after a follow-up period of 6 months was routinely recommended after successful recanalization of a CTO vessel in accordance with the guidelines for high-risk lesions^19^.

### Clinical endpoints and definitions

CTO was defined as a lesion with 100% stenosis and Thrombolysis In Myocardial Infarction (TIMI) flow grade 0 that exists for more than 3 months. The duration of occlusion was determined either based on the clinical record of previous coronary angiograms, clinical (onset of symptoms) or angiographic probability (e.g. collateralization).

Successful CTO PCI was defined as recanalization of the lesion with residual stenosis of ≤ 30% and TIMI flow grade 3, as assessed by visual estimation.

The primary endpoint of the retrospective analysis of our patient cohort study was defined as follows^20^:

1. *Re-occlusion* Defined as TIMI flow grade 0, as assessed by fluoroscopy of the treated vessel at the timepoint of surveillance coronary angiography
2. *Target vessel failure (TVF)* Defined as a combined endpoint by the presence of re-occlusion, restenosis, or target vessel revascularization (defined as a necessity for a repeated PCI within the former CTO vessel).

We also recorded procedural success rate, bleeding, vascular access complications requiring medical treatment and acute kidney injury (AKI). Bleeding was defined in accordance to the Academic Research Consortium. Type 3–5 bleedings were recorded. Acute kidney injury (AKI) was defined as an abrupt decrease in kidney function, according to the KDIGO AKI Guideline Work Group)^21^.

### Statistical analysis

Continuous normally distributed metric data are presented as mean and standard deviation and comparison between groups was performed using the Student’s *t* test; not normally distributed variables are presented as median and minimum and maximum values and compared using the Mann–Whitney U-Test. Categorial data are presented as absolute and relative frequencies and compared using the chi square test. A two-sided *p* value of < 0.05 was considered to be significant.

In order to have groups with similar baseline characteristics propensity score (PS) analysis was performed using a logistic regression model. In our model, age group was the dependent variable. We set cardiovascular risk factors (Diabetes mellitus, smoking, hypertension, hypercholesterolemia), chronic kidney disease (CKD; creatinine > 1.5 mg/dl), previous myocardial infarction (MI), previous coronary artery bypass grafting (CABG), previous stroke and multivessel disease as the independent variables in this model. After propensity score analysis we decided to use inverse probability of treatment weighting (IPTW) instead of PS matching method to avoid a reduction of the sample size and thus a loss of statistical power in our collective. With IPTW we balanced the distribution of covariates between the two patient groups. The evaluation of our collective takes place under consideration of the PS-weighting and makes the groups more comparable in respect to patient’s risk factors. PS is used to generate IPTW, which are then implemented in the analysis. IPTW analyses use the inverse of the propensity score as weights in patients who are over 80 years of age and the inverse of one minus the propensity score in patients under 80 years of age. Patients who had a low predicted probability of being aged > 80 years but were aged > 80 will be upweighted in the analysis, whereas patients with a high predicted probability of being aged > 80 who actually were aged > 80 will be down weighted in the analysis.

In addition, we calculated the effect size of the differences between groups to see if the results differ in a small, medium or large effect. Effect sizes between two means were interpreted as Cohens d (Cohens d < 0.5 small effect, 0.5–0.8 medium effect, > 0.8 large effect), effect sizes between two medians are interpreted as r (< 0.3 small effect, 0.3–0.5 medium effect, > 0.5 large effect), and for dichotomous variables (chi square test), Cramers V was used to calculate the effect size (< 0.1 small, < 0.3 medium, > 0.5 large effect). The statistical analyses was performed using SPSS (Version 23, IBM SPSS Statistics).

### Ethics approval

All data were obtained from individuals enrolled between 2016 and 2021 in the retrospective monocentric CTO PCI database of the University Medical Center in Mainz (DRKS #00025882), which was approved by the Ethics Committee of Rhineland Palatinate to be in accordance with the legal regulations and the declaration of Helsinki.

## Results

**Figure 1:**
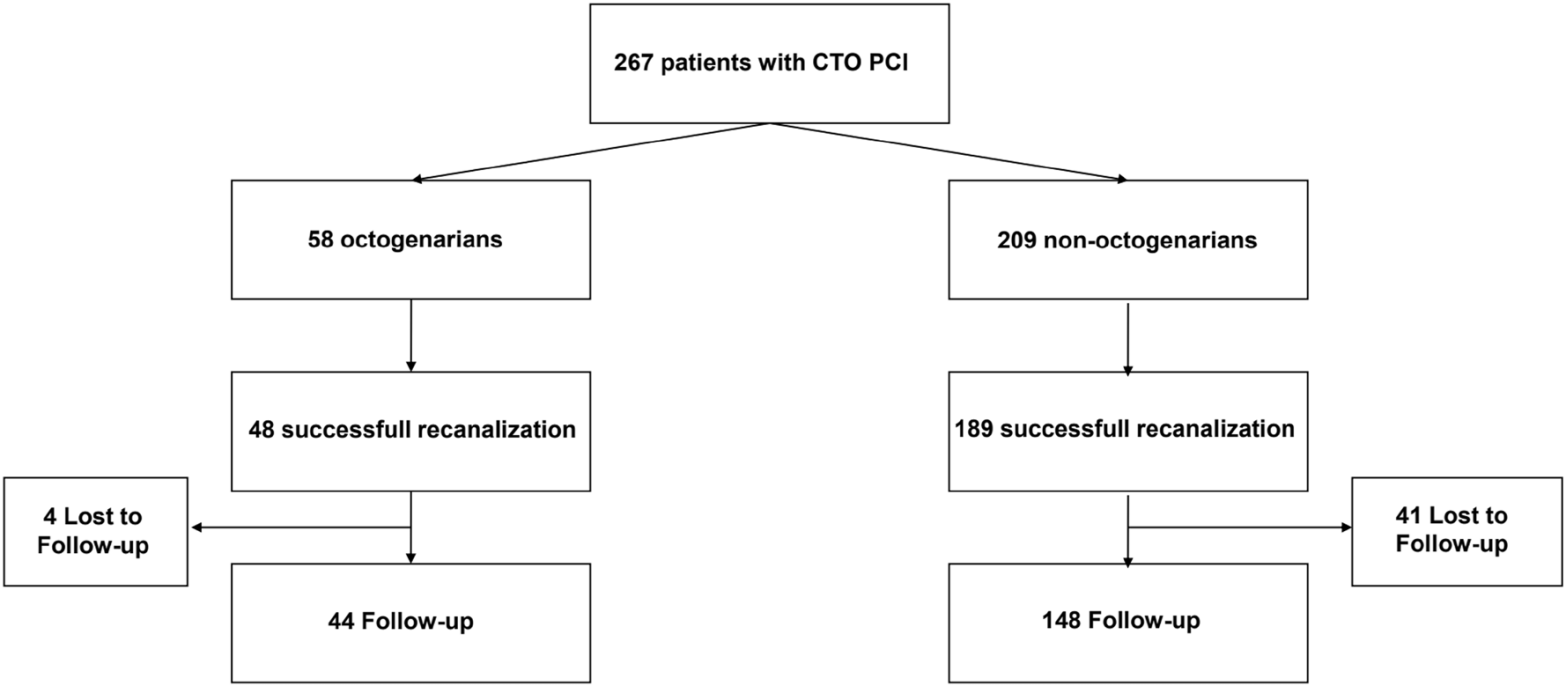
Study flow chart.

In total two hundred and sixty-seven patients of the CTO PCI database were included in the study, 58 patients over 80 years of age and 209 younger patients. Rate of successful CTO recanalization for all patients was 88.2%. Follow-up was available for 72% of the patients as shown in Fig. 1.

The median follow-up period was 219 ± 132 days. During this period, coronary angiography was performed to investigate the result after CTO recanalization. Clinical and angiographic parameters at baseline are shown in Table 1.

**Table 1:** Clinical and angiographic parameters at baseline.

	All patients (n = 267)	Octogenarians (n = 58)	Non-octogenarians (n = 209)	*p* value	Effect size
**Demographics characteristics**					
Age, years	68 (31–90)	82 (80–90)	65 (31–79)	**< 0.001**	
Male	221 (82.8)	43 (74.1)	178 (85.2)	**0.049**	0.12
BMI	28.61 ± 4.44	26.34 ± 3.27	29.25 ± 4.52	**< 0.001**	0.69
Diabetes mellitus	54 (20.2)	12 (20.7)	42 (20.1)	0.9	0.06
Hypertension	250 (93.6)	56 (96.6)	194 (92.8)	0.3	0.63
Hyperlipidemia	212 (79.4)	55 (94.8)	157 (75.1)	**0.01**	0.2
Current smoking	65 (24.3)	2 (3.4)	63 (30.1)	**< 0.001**	0.25
Ex-smoker	68 (25.5)	15 (25.9)	53 (25.4)	0.9	0.05
Multivessel CAD	228 (87.7)	54 (96.4)	174 (85.3)	**0.025**	0.13
CKD	29 (11)	15 (25.9)	14 (6.8)	**< 0.001**	0.25
LVEF	55 (20–65)	55 (27–55)	55 (20–65)	0.32	0.05
LVEF ≤ 40%	30 (11.6)	7 (12.5)	18 (8.9)	0.41	0.32
Previous stroke	12 (4.6)	5 (8.9)	7 (3.4)	0.08	0.1
PAD	36 (13.9)	9 (16.1)	27 (13.3)	0.59	0.03
Previous CABG	22 (8.5)	3 (5.4)	19 (9.4)	0.33	0.06
Previous MI	74 (28.5)	12 (21.4)	62 (30.4)	0.18	0.08
Previous PCI	186 (71.5)	44 (78.6)	142 (69.6)	0.18	0.08
NYHA	**1.89 ± 0.7**	**2.00 ± 0.79**	**1.86 ± 0.67**	**0.23**	**0.27**
CCS	**1.92 ± 0.7**	**1.94 ± 0.91**	**1.90 ± 0.73**	**0.73**	**0.05**
**Procedural characteristics**					
CTO vessel					
RCA	187 (70.0)	36 (62.1)	151 (72.2)	0.13	0.15
LAD	45 (16.5)	15 (25.9)	30 (14.4)	**0.03**	0.12
LCX	35 (13.5)	6 (10.3)	29 (13.9)	0.48	0.04
Procedural success	237 (88.2)	48 (82.8)	189 (90.4)	0.1	0.1
J-CTO score	1.82 ± 0.76	2 ± 0.72	1.77 ± 0.77	**0.04**	0.42
Antegrade access	235 (88.3)	55 (94.8)	180 (86.5)	0.08	0.1
Number of stents ≤ 3	96 (38.7)	16 (28.1)	80 (41.9)	0.06	0.11
Total stent length (mm)	52.13 ± 31.9	41.84 ± 28.21	54.99 ± 32.3	0.05	0.41
Fluoroscopic time (min)	34.31 ± 24.21	34.28 ± 24.9	34.32 ± 24.1	0.9	0.02
Contrast (ml)	240.5 ± 126.5	274.2 ± 159.6	308.5 ± 148.5	0.06	0.09
Hospital stay (days)	3 ± 5	3.7 ± 3	3.1 ± 5	0.29	0.14
**Complications at baseline**					
AKI	17 (6.6)	6 (10.3)	11 (5.3)	0.19	0.08
Bleeding	7 (2.7)	2 (3.5)	5 (2.4)	0.62	0.02
Aortic dissection	2 (0.8)	0	2 (1)	0.45	0.04
Ventricular fibrillation	3 (1.1)	0	3 (1.4)	0.36	0.05
complication of access side	4 (1.5)	1 (1.8)	3 (1.5)	0.87	0.01
Stroke	1 (0.4)	0	1 (0.5)	0.59	0.03
Cardiac death	3 (1.1)	2 (3.5)	1 (0.5)	0.06	0.11

Values are n (%), median (minimum and maximum), or mean ± SD.

*yrs* years, *CVD* cardiovascular disease, *BMI* body mass index, *CKD* chronic kidney disease, *LVEF* left ventricular ejection fraction, *PAD* peripheral artery disease, *CABG* coronary artery bypass graft, *MI* myocardial infarction, *PCI* percutaneous coronary intervention, *RCA* right coronary artery, *LAD* left anterior descending coronary artery, *LCX* left circumflex coronary artery, *AKI* acute kidney injury. Significant values are in [bold].

The main indication for CTO PCI was limiting physical complaints in everyday life. The complaints were assessed according to the New York Heart Association (NYHA) classification (dyspnoea) and Canadian Cardiovascular Society grading of angina pectoris (CCS). We found no difference in the NYHA classification between the octogenarian and non-octogenarian collective (*p* 0.23) or the CCS classification (*p* 0.57).

In 51 of 209 patients (24.4%) of the non-octogenarian group we had an additional prognostic indication with impaired left ventricular function (reduced LV-EF) or recurrent malignant heart rhythm disorders. In the octogenarian cohort 11 of 58 patients (19%) had a corresponding indication (*p* 0.38). We observed differences between our collectives in both the clinical and angiographic characteristics. Thus, the two collectives differed in the incidence of hyperlipidemia (94.8% in the octogenarians vs 75.1% in the non-octogenarian group, *p* = 0.01), CKD (25.9% vs 6.8%, *p* < 0.001), multivessel CVD (96.4% vs 85.3%, *p* = 0.025) and current smoking (3.4% vs 30.1%, *p* = 0.01). With regard to angiographic parameters, differences were seen in the CTO vessel, J-CTO Score (2.0 ± 0.72 vs 1.77 ± 0.77, *p* = 0.04) and total stent length (41.84 ± 28.21 vs 54.99 ± 32.3, *p* = 0.05). We found no statistical differences in the recorded in-hospital complications. One patient of the non-octogenarian collective had a stroke (0 vs 1 (0.5), *p* 0.59), and could be discharged without persisting neurological symptoms.

Three patients died after CTO-PCI (3.5% vs 0.5%, *p* = 0.06). An 80 year old patient with ischemic heart disease (LVEF of 35%), chronic kidney disease, previous stroke and PAD died 1 day after successful CTO PCI with observed ventricular fibrillation and unsuccessful resuscitation. An 85-year-old patient developed cardiogenic shock after CTO-PCI of the LAD. Twenty days after RCA-CTO-PCI, an 74-year-old patient died in septic shock with cardiac decompensation and renal failure.

Follow-up was available in 192 patients. Table 2 summarizes clinical and angiographic outcome at follow-up.

**Table 2:** Clinical and angiographic outcome at follow-up.

	All patients (n = 192)	Octogenarians (n = 44)	Non-octogenarians (n = 148)	*p* value	Effect size
**Angiographic outcome**					
Re-occlusion	29 (15.1)	0	5(3.4)	0.58	0.2
TVF	29 (15.1)	5 (11.3)	24 (16.3)	0.9	0.05
Acute MI	1 (0.5)	0	1 (0.7)	0.6	0.03
Major bleeding	2 (1.1)	0	2 (1.3)	0.5	0.04

Values are n (%).

*MACE* major adverse cardiac events, *TVF* target vessel failure, *MI* myocardial infarction.

In the follow-up period we could not detect any difference between the octogenarians and the younger patient cohort regarding the primary endpoint. There was only one case of acute MI due to late stent thrombosis. The other patients with TVF were identified by the routine control coronary angiography.

Table 3 presents baseline and follow-up parameters of the two weighted patient cohorts according to octogenarians and non-octogenarian patients.

**Table 3:** Baseline and outcome parameters of weighted groups according to octogenarians and non-octogenarians.

	All patients (n = 248)	Octogenarians (n = 118)	Non-octogenarians (n = 130)	*p* value	Effect size
**Demographics characteristics**					
Age, years	78 (31–90)	82 (80–90)	66 (31–79)	**< 0.001**	
Male	181 (72.8)	70 (49)	111 (85.4)	**< 0.001**	0.29
BMI	28.3 ± 4.06	28.1 ± 3.7	28.5 ± 4.4	0.35	0.11
Diabetes mellitus	45 (18.1)	18 (15.1)	27 (20.9)	0.26	0.07
Hypertension	237 (95.4)	115 (97.4)	122 (93.6)	0.16	0.08
Hyperlipidemia	211 (85.1)	111 (94.2)	100 (76.9)	**< 0.001**	0.24
Current smoking	41 (16.6)	3 (2.3)	38 (29.6)	**< 0.001**	0.35
Ex-smoker	58 (23.5)	25 (20.9)	34 (25.9)	0.35	0.05
Multivessel CVD	222 (89.5)	113 (96.3)	114 (86.9)	0.06	0.22
CKD	29 (11.7)	14 (11.6)	15 (11.8)	0.9	0.05
LVEF	55 (20–65)	55 (27–55)	55 (20–65)	0.8	0.01
LVEF ≤ 40%	23 (9.3)	12 (9.9)	11 (8.7)	0.65	0.02
Previous stroke	13 (5.1)	6 (5.3)	6 (4.9)	0.86	0.01
PAD	37 (14.9)	20 (16.6)	17 (13.4)	0.39	0.05
Previous CABG	17 (7)	6 (5.1)	11 (8.8)	0.29	0.06
Previous MI	74( 29.8)	38 (31.8)	36 (27.4)	0.43	0.04
Previous PCI	183 (73.7)	89 (75.2)	94 (72.4)	0.57	0.03
NYHA	**1.93 ± 0.69**	**1.95 ± 0.72**	**1.87 ± 0.67**	**0.19**	**0.17**
CCS	**1.95 ± 0.79**	**1.90 ± 0.72**	**2.00 ± 0.87**	**0.34**	**0.12**
**Procedural characteristics**					
CTO vessel					
RCA	149 (60.3)	60 (50.9)	89 (68.7)	0.05	0.18
LAD	52 (20.9)	30 (25.6)	22 (16.6)	0.1	0.10
LCX	46 (18.7)	27 (22.7)	20 (15.1)	0.12	0.09
Procedural success	229 (92.3)	106 (90.2)	123 (94.2)	0.23	0.07
J-CTO Score	1.8 ± 0.77	1.98 ± 0.67	1.64 ± 0.82	**< 0.001**	0.47
Antegrade access	226 (91.5)	115 (97.4)	112 (86.1)	**0.02**	0.16
Number of stents ≤ 3	103 (42.7)	53 (45)	50 (40.5)	0.47	0.04
Total stent length (mm)	51.6 ± 28.6	46.93 ± 24.67	55.82 ± 31.2	**0.01**	0.31
Fluoroscopic time (min)	32.52 ± 27.5	29.82 ± 23.81	35.12 ± 30.58	0.13	0.19
Contrast agent(ml)	270.3 ± 159.6	253.9 ± 203.9	300 ± 151	**0.01**	0.22
Hospital stay (days)	3.5 ± 4.7	3.9 ± 3.5	3.1 ± 5.5	0.15	0.2
**Complications at baseline**					
AKI	20 (8.3)	12 (9.9)	9 (6.7)	0.37	0.05
Bleeding	7 (2.9)	2 (1.9)	5 (3.8)	0.3	0.06
Aortic dissection	1 (0.4)	0	1 (0.8)	0.33	0.06
Ventricular fibrillation	2 (0.7)	0	2 (1.3)	0.17	0.08
complication of access side	7 (2.8)	5 (4.4)	2 (1.4)	0.2	0.08
Stroke	1 (0.2)	0	1 (0.4)	0.34	0.34
Cardiac death	3 (1.2)	2 (1.7)	1 (0.6)	0.5	0.04

	All patients (n = 137)	Octogenarians (n = 46)	Non-octogenarians (n = 91)	*p* value	
**Outcome parameters**					
Re-occlusion	3 (2.2)	0	3 (3.3)	0.24	0.09
TVF	21 (15.4)	6 (12)	16 (17.2)	0.46	0.06
Acute MI	1 (0.7)	0	1 (1.4)	0.49	0.05
Major bleeding	2 (1.4)	0	1 (1.4)	0.48	0.05

Values are n (%), median (minimum and maximum), or mean ± SD.

*yrs* years, *CVD* cardiovascular disease, *BMI* body mass index, *CKD* chronic kidney disease, *LVEF* left ventricular ejection fraction, *PAD* peripheral artery disease, *CABG* coronary artery bypass graft, *MI* myocardial infarction, *PCI* percutaneous coronary intervention, *RCA* right coronary artery, *LAD* left anterior descending coronary artery, *LCX* left circumflex coronary artery, *AKI* acute kidney injury, Values are n (%), *MACE* major adverse cardiac events, *TVF* target vessel failure, *MI* myocardial infarction. Significant values are in [bold].

Even after calculation of the effect size we still found comparable values for success rate and complication. All parameters showed only a small effect size of the differences which is presented in Tables 1, 2 and 3.

### Inverse-probability-of-treatment weighting analysis

After IPTW adjustment, the covariates in the two groups were similar, except that gender (< 0.001) and smoking (< 0.001) remained higher in the collective under 80 years. Before IPTW adjustment by comparison the octogenarians with non-octogenarians CTO PCI was associated with a low risk of complications in both collectives. There was no difference in the outcome parameters. In the IPTW analysis we still found no difference in Re-occlusion (*p* 0.24), TVF (*p* 0.46), acute MI (*p* 0.49) and major bleeding (*p* 0.48). Investigated events were still low in both groups with no significant differences.

## Discussion

Our study investigated feasibility, complications and clinical outcome of CTO revascularization in patients older than 80 years in comparison to younger patients. We could demonstrate that angiographic success of CTO revascularization was comparable between both cohorts. We could not detect any difference in the occurrence of periprocedural complications or major cardiovascular events in the clinical course as well as over the complete follow-up period in the octogenarian cohort.

There is limited evidence regarding treatment of CAD and outcome in octogenarians undergoing PCI. The knowledge mainly rises from registry or observational single-center studies; prospective randomized trials are rare. In an analysis of the National Inpatient Sampling database in 11.056.559 patients who underwent PCI, a higher rate of in-hospital mortality, cardiopulmonary complications, postprocedural stroke, acute kidney injury and postprocedural thromboembolic complications after PCI in octogenarians compared to patients < 80 years of age was demonstrated^22^. Yet, several studies have shown an improvement in life expectancy in elderly patients with CAD and ACS treated with PCI compared to OMT^1,23–26^. Based on the current evidence, the benefit of PCI over OMT in elderly patients is still controversial^23^. Thus, the decision in favor of PCI remains on an individual basis in elderly patients, weighting the risks of complications, frailty and benefits^24^. Recommendations regarding the optimal treatment of elderly patients with CTO are even more unclear. Most evidence of CTO PCI in elderly is derived from studies with rather small patients cohorts. These studies demonstrated that CTO PCI is feasible and beneficial in the patient group > 75 years. However, the average age of these collectives was ≤ 80 years^8–13^. To our knowledge, we have currently examined the collective with CTO with the highest age in average.

Previous studies demonstrated a high success rate of CTO PCI, which did not differ between younger and older patients (≥ 75 years)^9^. The Euro CTO registry reported a success rate of 82% in patients ≥ 75 years^7^. These results support our findings of a high overall success rate in our collective (88.2%). The rate of success was numerically lower in the patient group older than 80 years, but we did not find a statistically significant difference here. We explain this to the more complex CTO lesions in the octogenarian group with a significantly higher J-CTO score (octogenarians: 2 ± 0.72 vs non-octogenarians: 1.77 ± 0.77, *p* value: 0.04) and the smaller sample size of the octogenarian collective.

Rotational atherectomy was used in 8 of the 267 patients (2.99%), 8 of 209 patients (3.8%) of the non-octogenarian group and no patient of the octogenarian collective (*p* 0.5).

A higher rate of procedural complications and in-hospital MACE is expected in the octogenarian. Toma et al. found no difference in their collective. There was no differences in MACE (1% vs 0.9%, p 0.564), death (0.5% vs 0.4%, *p* = 0.577), MI (0.5% vs 0.4%, *p* = 0.577), TVR (0.2% vs 0.2%, *p* = 0.599), coronary perforation (0.5% vs 0.3%, *p* = 0.2719, cardiac tamponade (0. 5% vs 0.5%, *p* = 0.665), cerebrovascular accident (0.2% vs 0.1%, *p* = 0.367), surgical repair (0.7% vs 0.5%, *p* = 0.212), vascular complication (0.1% vs 0%, *p* = 0.633), bleeding requiring transfusion (2.2% vs 0.2%, *p* ≤ 0.001)^15^. A single-center study from Japan observed that CTO PCI could be safely performed in patients older than 75 years with a low rate of intra-hospital complications14. Another study from the People's Republic of China compared an elderly group (age ≥ 75 years, n = 68) and non-elderly group (age < 75 years, n = 178) and found a comparable rate of intra-hospital MACE, vascular access complication and major bleeding^8^. Our study is therefore in good agreement with previous reports in terms of complication rates. Our experience demonstrates a low rate of intra-hospital complications. AKI was the most common intra-hospital complication in our octogenarian collective, none of our patients developed a need for dialysis.

In addition to multiple comorbidities and an increased risk of complications, octogenarians are more likely to have complex and calcified lesions^4^. Optimal stent implantation and expansion is complicated by coronary calcification. This may increase the risk of restenosis in elderly^27^. For these reasons, it is questionable whether CTO PCI is beneficial in this cohort, as CTO lesions are often heavily calcified and have a long lesion length. There is currently no data available on the restenosis rate of octogenarians after CTO PCI. In our collective, both groups showed a comparable restenosis rate with a non-significant trend to fewer events in octogenarians (5 (11.3%) vs 24 (16.3%), *p* 0.90).

Other important factors that determine whether elderly patients benefit from CTO PCI are quality of life and event-free long-term outcome^9–15^. Over an average follow-up period of 2.3 years. Toma et al.^15^ showed a survival benefit in both younger and older patients. After 3 years of follow-up, Tanaka et al.^14^ showed a better cardiac survival in older patients with successful CTO PCI than those with failed PCI (97.6% vs 76.9%, *p* = 0.005). We therefore assessed cardiovascular ischemic outcomes as well. During our follow-up period of 219 ± 132 days we observed one acute MI caused by a late stent thrombosis in the group of non-octogenarians and 29 TVF (11.3% vs 16.3%, *p* 0.90).

In our follow-up period, we did not observe any additional deaths or stroke, except these intra-hospital complications. For the primary endpoint we found no difference between the two groups. Re-occlusion and TVF were numerically lower in the group of octogenarians but not statistically significant.

Our study results show that CTO PCI can safely be offered to patients older than 80 years. However, until there are data from large randomized trials, it should be an individual treatment decision. In particular, the frailty of the patient must be taken into account.

### Study limitations

We retrospectively analyzed data from a single center database. Our data cannot be compared to other therapy strategies (such as OMT or CABG). The possible effects should be investigated on a larger group size, which can best be achieved through multicentric recruitment. Our analysis is hypothesis generating, further prospective studies are needed to investigate the benefit of CTO PCI in octogenarians.

## Conclusion

Our study is the first investigating clinical and angiographic outcome after successful recanalization of CTO lesions in octogenarians. We found a high success rate of complete recanalization of CTO lesions and low rate of complications for patients over 80 years of age. Comparing these endpoints with the under-80 years collective, we found no difference between the two collectives. Our results suggest that CTO PCI is feasible and safe even for older patients.
